# Frequency conversion of microwave signal without direct bias current using nanoscale magnetic tunnel junctions

**DOI:** 10.1038/s41598-018-37415-8

**Published:** 2019-01-29

**Authors:** J. M. Algarin, B. Ramaswamy, I. N. Weinberg, Y. J. Chen, I. N. Krivorotov, J. A. Katine, B. Shapiro, E. Waks

**Affiliations:** 1 Institute for Research in Electronics and Applied Physics (IREAP), University of Maryland, College Park, Maryland 20742 United States; 2 Fischell Department of Bioengineering, University of Maryland, College Park, Maryland 20742 United States; 3 Weinberg Medical Physics Inc., North Bethesda, Maryland 20852 United States; 4 Department of Physics and Astronomy, University of California, Irvine, California 92697 United States; 5 HGST Research Center, San Jose, California 95135 United States; 6 Institute for Systems Research (ISR), University of Maryland, College Park, Maryland 20742 United States

## Abstract

Frequency conversion forms an integral block of the electronic circuits used in various applications including energy harvesting, communications and signal processing. These frequency conversion units however require external power sources and occupy a large device footprint making it difficult to be integrated in micro-circuits. Here we demonstrate that nanoscale magnetic tunnel junctions can act as frequency converters without an external power supply or DC bias source. The device directly mixes an external microwave signal with the internal spin precession oscillations to create new frequencies tunable by an external magnetic field in a single device with a small device footprint. We observe up-conversion and down-conversion of the input signal for excitation frequencies between 2 GHz and 6 GHz. We also show that the device acts as a zero-bias rectifier that can generate voltages exceeding 12 mV when the excitation frequency matches the natural oscillations mode of the device.

## Introduction

The ability to perform signal frequency conversion plays an important role in various applications that include communication, analog circuits, sensing, and power electronics. For example, in superheterodyne receivers, microwave circuits convert the frequency of received signal to an intermediate frequency for processing^1^. In Radio Frequency Identification (RFID) technology, which has a wide range of advantages in the global supply chain^2^, a primary source delivers power to a receiver system called a tag in the form of radio frequencies^3^. Using a rectifier, the tags converts these radio frequencies to a DC voltage that powers the circuits in the device^3^. Bionic implants and low power sensor networks use similar rectification circuits^4^.

Currently, frequency conversion is challenging for applications involving low incident power levels. Most rectifier circuits require a minimum threshold voltage which leads to an unresponsive dead zone at low input voltage amplitudes^5^. Low-voltage applications therefore require additional amplifying circuits to reduce the dead zone threshold. But amplifiers typically require external power sources and are therefore difficult to implement in passive applications that do not have access to onboard power sources. Moreover, with the advent of micro-scale or nanoscale circuits to reduce the device footprint, compatibility with miniaturization is an important requirement for frequency converters used in various applications. For these applications, a single nanoscale device that can perform frequency conversion without external power would be extremely desirable.

Nanoscale magnetic tunnel junctions provide an alternate approach to achieve frequency conversion or rectification of low amplitude signals in a nanoscale device footprint. These nano-oscillators take as their input direct currents and convert them to microwave current oscillations^6–10^. Alternatively, they can perform frequency modulation when an additional microwave frequency signal is applied to the device. Pufall *et al*. originally demonstrated frequency modulation in a nanoscale magnetic tunnel junctions using a modulation signal of 40 MHz^11^. Subsequent work provided the first experimental evidence of frequency modulation between an external signal and the microwave signal oscillations of the device, with modulation frequencies of up to 3.2 GHz^12–14^. However, these works required an external power supply to inject direct current to the device that generated microwave signal oscillations through nanoscale magnetic tunnel junctions. Whether such devices can attain frequency conversion passively without a dc bias current remained an important open question.

Here, we report frequency conversion and rectification in nanoscale magnetic tunnel junction without a dc bias current. By injecting microwave signal wirelessly into the device, we show that it can generate both an up-converted and down-converted frequency signal that is tunable by an external magnetic field. We also show that the device acts as a passive microwave rectifier for low input power levels, and is therefore compatible with low voltage applications. These results open spintronic devices as potential passive frequency converters that may find important applications in signal processing, energy harvesting, and sensing.

## Results

**Figure 1:**
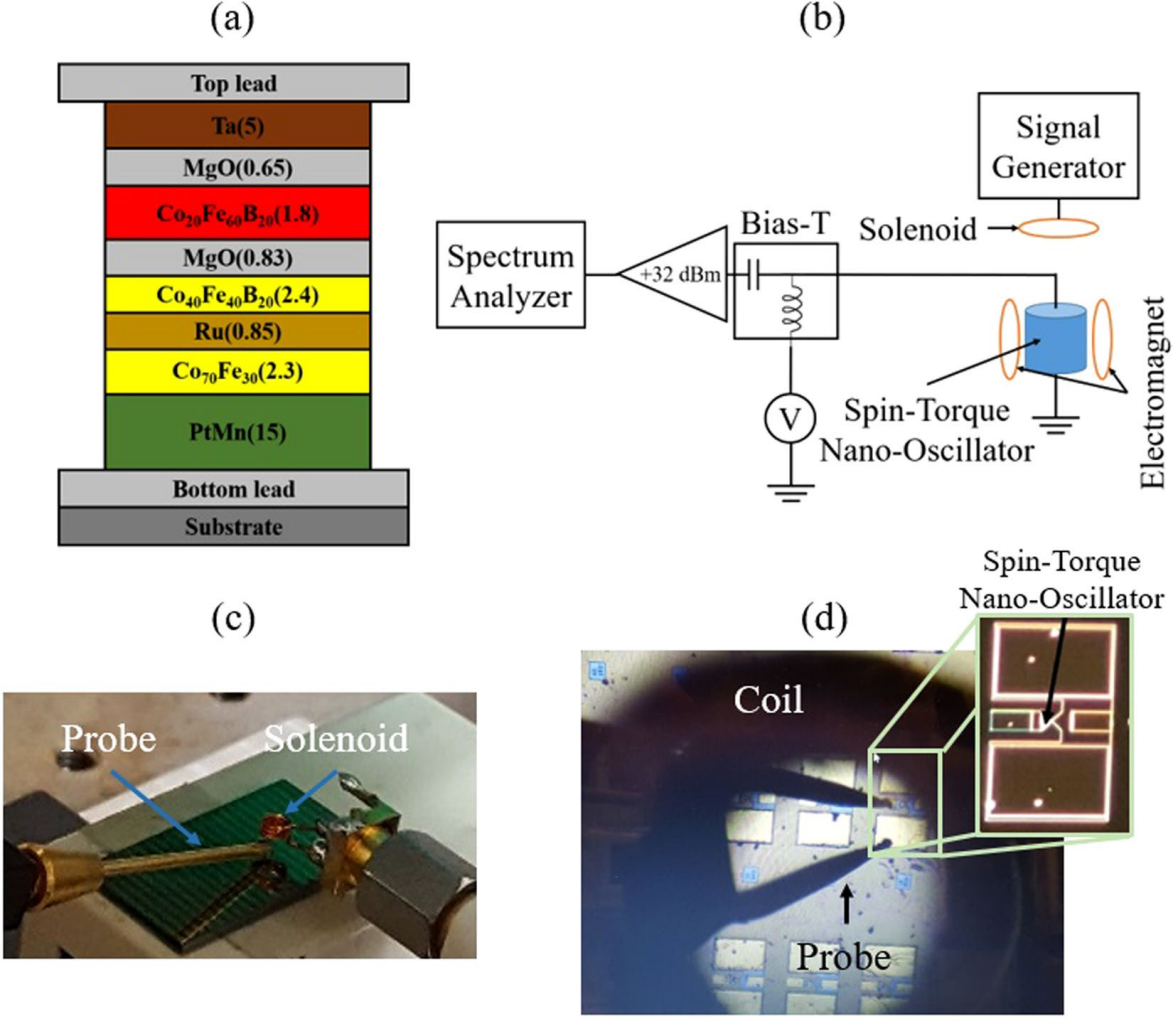
(**a**) Schematic of the nanopillar nanoscale magnetic tunnel junction device. The numbers in parenthesis are the layer thicknesses in units of nanometers. (**b**) A schematic of the microwave circuit used for power spectral density and direct voltage measurement from the device. (**c**) Picture of the setup showing the nanoscale magnetic tunnel junction chip and the solenoid. (**d**) Magnification of the nanoscale magnetic tunnel junction connected to the microprobe with the coil above. The microprobe and the connection pads along with the nanoscale magnetic tunnel junction forming an effective coupler.

To perform frequency conversion, we utilize the experimental configuration illustrated in Fig. 1(b). We position a solenoid antenna directly above the device surface as shown in Fig. 1(c,d). The solenoid has three turns with 1 mm diameter and 1.2 mm length fabricated with copper wire of 0.4 mm diameter. We input microwave power into the solenoid using a microwave signal generator (Agilent E8257D). The solenoid transmits the microwave power wirelessly to the device through electromagnetic coupling with the coplanar electrodes attached to the device (see inset of Fig. 1(d)). Then the solenoid wirelessly induces a microwave current that flows through the magnetic tunnel junction. The input signal frequency is 3.5 GHz that produces maximum transmission from the solenoid to the device. The solenoid also produces a weak microwave magnetic field mainly orientated perpendicular to the device surface. Simultaneously to the microwave power input to the device, we use an electromagnet (GMW associates) to apply an external magnetic field along the hard in-plane axis. The microwave power input simultaneous to the external magnetic field results in the precession of the magnetic free-layer^15^ with maximum resistance oscillations. The free layer precession in combination with the induced microwave signal generates an electromagnetic signal at microwave frequency across the oscillator terminals. We detect the signal from the device using a non-magnetic picoprobe (10-50/30-125-BeCu-2-R-200, GGB industries). We use a bias tee (Pasternack, PE1604) to extract the microwave signal at the output of the device using the capacitive port. A low noise amplifier (Pasternack PE15A3005, gain = 32 dB and input impedance = 50 Ω) amplifies the output from the device. We analyze the amplified output using a spectrum analyzer (Agilent 8564 EC). A measurement of the transmission between signal generator to the spectrum analyzer shows a local maximum of −20 dB at 3.5 GHz. Additionally, we use the bias tee to measure the direct voltage component of the signal generated by the device using the inductive port.

**Figure 2:**
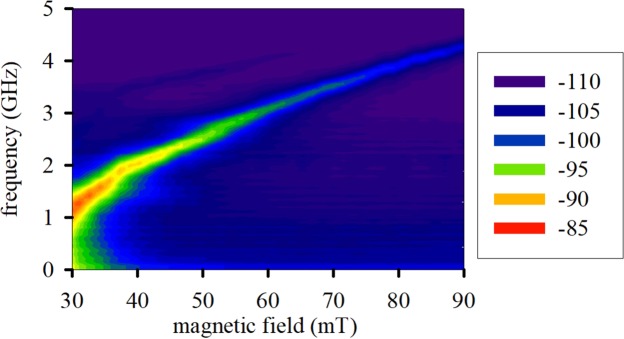
Power spectral density measured in dBm of the signal from the nanoscale magnetic tunnel junction for a direct current of 100 μA.

We first characterize the nanoscale magnetic tunnel junction (Fig. 1(a)) by injecting a constant input current to determine its spectral output. We input the constant current with an external power supply and monitor the microwave output power using a microwave spectrum analyzer. Figure 2 shows the power spectral density of the device output as a function of magnetic field, where we apply a magnetic field along the in-plane (hard) axis and inject an input current of 100 µA. The mean oscillation mode has a positive magnetic field tunability of 0.1 GHz/mT. We also observe a second order oscillation mode at higher frequency, but its amplitude is about 20 dB smaller than the amplitude of the main mode.

**Figure 3:**
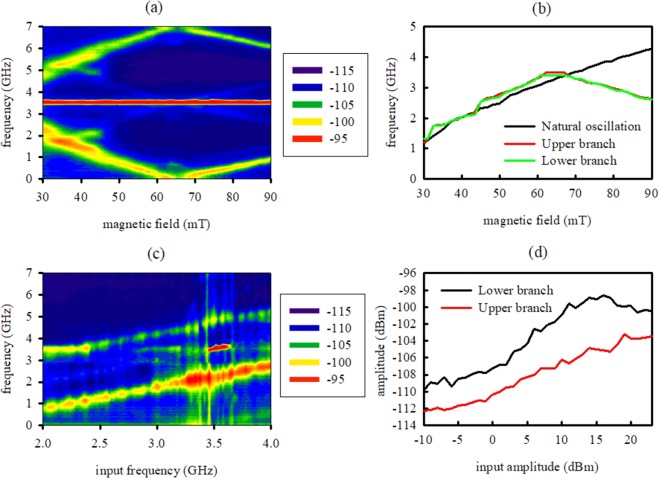
(**a**) Microwave output spectrum of the device measured in dBm as a function of applied magnetic field. (**b**) Frequency difference between the upper (red line) and lower (green line) branch and the excitation frequency, along with the natural frequency (black line) of the device (**c**) Power spectral output at 31 mT and 23 dBm for different excitation frequencies. (**d**) Peak power for the lower (black line) and upper (red line) branch for 31 mT and 3.5 GHz at different excitation amplitudes.

Next, we remove the DC current and operate the device at zero bias. Table 1 summarizes the experiments performed in this paper. We first wirelessly inject a microwave input current at 3.5 GHz to the device through the solenoid (Experiment I). We excite the solenoid using an input power of 23 dBm, and place it a distance of 0.5 mm from the device. The solenoid induced microwave current but it also creates a microwave magnetic field that add up to the static magnetic field. However, for input power of 23 dBm the magnitude of the microwave magnetic field is on the order of microteslas, a negligible magnetic field compared to the static magnetic field of tens of millitesas. Figure 3(a) shows the measured microwave output spectrum of the device as a function of applied static magnetic field. We observe a signal at the excitation frequency, along with two branches corresponding to up-converted and down-converted frequencies. The two branches lie symmetrically about the excitation frequency. Figure 3(b) plots the frequency difference between the upper and lower branch and the excitation frequency, along with the natural frequency of the device measured in Fig. 2. The frequency differences completely overlap with the natural frequency of the device suggesting that they are induced by mixing between the wirelessly induced microwave signal and the natural device oscillation mode, as expected from the concepts described on Methods section. While the lower branch can be explained due to the frequency mixing of the natural device oscillations mode and the input microwave signal, the higher branch comes back down when external magnetic field is higher than 66 mT, this effect cannot be explained by the same frequency mixing. If the free and pinned layer magnetizations of the nanoscale magnetic tunnel junction device are nearly collinear, there will be also resistance oscillations at a frequency equal to double frequency of natural oscillation mode that will create additional branches. Another possible explanation is that a higher order oscillation mode from the signal generator is efficiently transmitted by the solenoid to the device. Because some components in the setup, like the bias-tee, are limited to frequencies smaller than 7 GHz, we performed numerical simulations of the solenoid in CST Microwave Studio (Computer Simulation Technology Inc.). Numerical simulation shows a maximum transmission of microwave signals at 11 GHz, close to the third order oscillation mode of the signal generator (10.5 GHz). We note that we obtain these mixed signals without an external bias current. We generate all signals by wirelessly exciting the device with microwave.

**Table 1:** Summary of experiments performed in this work.

Experiment	Magnetic Field	Input Frequency	Input Amplitude	Distance	Measurement	Figure
I	X	3.5 GHz	23 dBm	0.5 mm	PSD	3(a,b)
II	31 mT	X	23 dBm	0.5 mm	PSD	3(c)
III	31 mT	3.5 GHz	X	0.5 mm	PSD	3(d)
IV	X	3.5 GHz	23 dBm	0.5 mm	Rectified Voltage	4(a)
V	66 mT	3.5 GHz	23 dBm	X	Rectified Voltage	4(b)

“X” indicates the magnitude that we sweep in the given experiment. The magnitudes are the external magnetic field produced by the electromagnet, the input signal frequency, the input signal amplitude and the distance between the solenoid and the device. We also indicate the measured magnitude (power spectral density (PSD) or rectified voltage) and the corresponding figure.

To investigate the dependence of the frequency mixing effect, we sweep the frequency of the microwave signal wirelessly injected into the solenoid (Experiment II). Figure 3(c) shows the spectral output as a function of the input frequency, where we use an input power of 23 dBm to the solenoid and an external magnetic field of 31 mT. As we sweep the input frequency, we observe a shift of the sidebands. The frequency converted sidebands are always symmetrical with respect to the input frequency and their difference equals the natural frequency of the device (1.5 GHz at 31 mT). This measurement supports the assertion that the sidebands are produced by frequency mixing of the natural device oscillation with the input microwave signal. The sideband powers are maximal when the carrier frequency is close to 3.5 GHz, which corresponds to the maximum transmission frequency between the solenoid and the device. While the sideband powers changes at different frequencies as a consequence of the transmission profile, the sidebands are present for any input frequency, demonstrating the broadband nature of the frequency conversion process.

We next study the dependence of the frequency conversion process as a function of microwave input power (Experiment III). Figure 3(d) shows the output power at the maximum of the two sidebands measured from the spectral output at different input powers when the excitation frequency is 3.5 GHz and the external magnetic field is 31 mT. The power of the two sidebands increases gradually with input power amplitude and persists even at input powers below −10 dBm. For input power of −10 dBm to the solenoid, we estimated the microwave power induced in the nanoscale magnetic tunnel junction was −21 dBm. These results demonstrate that the frequency mixing process can operate at extremely low input powers.

**Figure 4:**
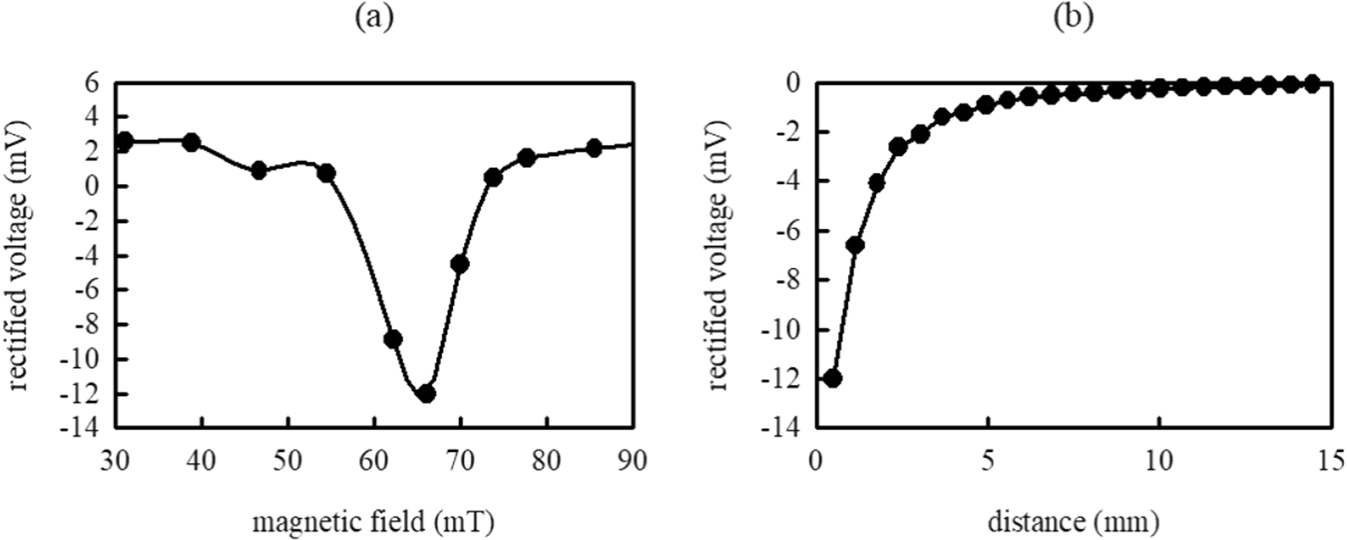
(**a**) Rectified voltage at different external magnetic fields. (**b**) Rectified voltage for 66 mT and different distances between the solenoid antenna and the spintronic device.

In Fig. 3(a), we see that at 66 mT the lower branch reaches DC frequencies because the signal frequency matches with the free layer oscillations frequency^16^. At this operating condition, the device can behave as a bias-free rectifier that converts microwave signals to DC voltages. Figure 4(a) shows the direct voltage measured across the device as a function of external magnetic fields using an input signal of 23 dBm (Experiment IV). At lower magnetic fields we attain a broadband rectification of 2 mV, but at 66 mT we attain a strong negative voltage of −12 mV. We obtained similar results when removing the solenoid for wireless excitation and exiting the device by direct microwave current injection. In both cases the device breakdown was the limiting factor that prevented us from achieving higher voltages. Figure 4(b) shows the amplitude of the rectified voltage as a function of distances between the solenoid and the device (Experiment V). The direct voltage decreases with increasing distance between the solenoid and the device. We observe a direct voltage produced in the device for wireless excitation from distances up to 15 mm.

## Discussion

In this work we have demonstrated that nanoscale magnetic tunnel junctions can act as low-power bias-free frequency mixers and rectifiers. The device converts frequencies by mixing them with internal resistance oscillation modes, producing up-converted and down converted frequencies symmetrically across the carrier. We can tune the generated signal frequencies by either changing the carrier or the external magnetic field applied to the device. At the condition where the input frequency matches the internal oscillation frequency of the device we attain a bias-free voltage rectifier that can operate at very low voltage levels. The device also enables wireless operation, which may have important beneficial properties for wireless sensing and energy transfer. We employed an electromagnet to produce static magnetic field to tune the device resistance oscillations. However, we obtained similar results with similar devices but employing a permanent magnet that do not need any external power supplier (see Supplementary Material). Also devices having a planar polarizer and a perpendicular free layer could produce resistance oscillations in the absence of external magnetic field^16^ that can be important to practical applications.

There are two possible explanations to the frequency mixing behavior that we observe in this work. A first explanation comes from the insight provided by Tulapurkar *et al*.^17^, who demonstrated that the application of a small microwave alternating current to a nanoscale magnetic tunnel junction can excite resistance oscillations. Microwave input oscillations together with resistance oscillations picks up a constant output voltage^17,18^. This DC constant voltage can subsequently induce resistance oscillations at the natural frequency of the device through spin transfer torque, which can subsequently mix with the input signal to create up-converted and down-converted branches. However, as shown in Fig. 4(a), the device produced a strong DC voltage at 66 mT, while Fig. 3(a) shows that frequency conversion happens at any external magnetic field. A second explanation comes from Zhou work^19^. This work shows thermally excited ferromagnetic resonance, also called mag-noise, that produces resistance oscillations even without input DC current. The thermally excited resistance oscillations can mix with the input signal to create up-converted and down-converted branches.

We could further increase the efficiency of the device by employing device geometries that exhibit larger resistance oscillations^15,20^. In our current setup, we induce the signal by wireless electromagnetic coupling between the solenoid directly connected to the signal generator and the wiring surrounding the device like copper pads or picoprobe. We do not optimize the wireless excitation method in terms of impedance matching and we do not have a receiver antenna on-chip, which leads to poor coupling. We could significantly improve the coupling and impedance matching by utilizing a matching network and better antenna geometries. Previous works applied wireless microwave excitation to magnetic tunnel junctions^21,22^, but there is an important difference between our work and these past results. Previous works employed the wireless microwave field to excite magnetization directly, while in our work we employ the microwave field to inductively excite microwave current through the device and induce mixing between thermally excited ferromagnetic resonance and the induced microwave current. This distinct difference from the previous experiment allowed us to observe wirelessly induced frequency up-conversion and down-conversion without a dc bias current.

Ultimately, in this work we achieve a proof-of-concept demonstration of the physics that shows how the device can act as a mixer and rectifier without external dc bias current. Also, our results present an alternative approach to wirelessly excite the nanoscale magnetic tunnel junction^21,22^ and enable it to function as a frequency converter or a rectifier that may have applications in signal processing, energy harvesting and *in-vivo* bioelectric stimulation or modulation.

## Methods

### Fabrication

The device that we employ in this work is an elliptical magnetic tunnel junction nanopillar with lateral dimensions 60 nm · 130 nm. Figure 1a shows the complete layer structure for the device, with thicknesses indicated in parentheses in units of nanometers. We deposited all layers using magnetron sputtering in a Singulus TIMARIS system, and patterned the magnetic tunnel junctions using electron beam lithography followed by ion milling. The synthetic antiferromagnet is PtMn(15)/Co_70_Fe_30_(2.3)/Ru(0.85)/Co_40_Fe_40_B_20_(2.4) with the Co_70_Fe_30_ pinned layer and the Co_40_Fe_40_B_20_ reference layer antiferromagnetically coupled by the tuned thickness of Ru. Prior to patterning, we anneal the multilayer for 2 hours at 300 °C in a 1 T in-plane field to set the pinned layer exchange bias direction parallel to the long axis of the nanopillars.

### Concepts on frequency conversion

We excite the magnetic tunnel junction with an input signal frequency *f*_*rf*_ given by

1 $${I}_{rf}=I\,\sin (2\pi {f}_{rf}t)$$

Where *I* is the amplitude of the wirelessly induced microwave current. This input signal excites resistance oscillations in the magnetic tunnel junction at frequency *f*_*MTJ*_. In a first approximation, we can write the device resistance as

2 $$R(t)={R}_{0}+{\rm{\Delta }}R\,\sin (2\pi {f}_{MTJ}t)$$

Where *R*_0_ is the resistance for zero input microwave signal, and Δ*R* is the dynamic range of the resistance when microwave signal in flowing through the device. Then, the induced voltage across the device terminals is given by the Ohms law, resulting in

3 $${V}_{MTJ}(t)={I}_{rf}{R}_{0}\,\sin (2\pi {f}_{rf}t)+I{\rm{\Delta }}R\,\sin (2\pi {f}_{rf}t)\sin (2\pi {f}_{MTJ}t)$$

Then, taking into account the trigonometric identity 2·$\sin \,A\cdot \,\sin \,B=\,\cos (A-B)-\,\cos (A+B)$, we can rewrite Eq. (3) as

4 $${V}_{MTJ}(t)={I}_{rf}{R}_{0}\,\sin (2\pi {f}_{rf}t)+\frac{I{\rm{\Delta }}R}{2}\,\cos (2\pi ({f}_{rf}-{f}_{MTJ})t)-\frac{I{\rm{\Delta }}R}{2}\,\cos (2\pi ({f}_{rf}+{f}_{MTJ})t)$$

Eq. (4) shows that the excitation of resistance oscillations in the magnetic tunnel junction produces up and down frequency conversion. The second term on the right side of Eq. (4) shows that the down frequency oscillations happens at frequency ${f}_{rf}-{f}_{MTJ}$ or ${f}_{MTJ}-{f}_{rf}$ depending on which frequency is higher. If ${f}_{rf}={f}_{MTJ}$, the second term in the right side of Eq. (4) indicates that a rectified voltage rises with amplitude *I*Δ*R*/2. Third term on the right side of Eq. (4) shows that upper frequency oscillations happens at ${f}_{rf}+{f}_{MTJ}$.

## Supplementary information

Results from permanent magnet

Dataset 1

Dataset 2A

Dataset 2B

Dataset 2C

Dataset 2D

Dataset 3A

Dataset 3B

## Data Availability

The datasets generated during and/or analyzed during the current study are included in this published article (see Supplementary Material).
