# Supplementary material for: Frequency conversion of microwave signal without direct bias current using nanoscale magnetic tunnel junctions
Source: Sci Rep. 2019 Jan 29;9:828. doi: 10.1038/s41598-018-37415-8 (PMC6351604; doi:10.1038/s41598-018-37415-8)
Supplement: Supplementary file 1 — Results from permanent magnet [file 41598_2018_37415_MOESM1_ESM.docx]

**Frequency conversion of microwave signal without direct bias current using nanoscale magnetic tunnel junctions.**

J.M. Algarin^1^, B. Ramaswamy^2^, I.N. Weinberg^3^, Y.J. Chen^4^, I.N. Krivorotov^4^, J. A. Katine^5^, B. Shapiro^2,6^, and E. Waks^1^

Supplementary Material I: Results from permanent magnet.

As we mention in the discussion section, we also obtained up-conversion and down-conversion results with similar devices but employing a permanent magnet. With a permanent magnet we do not need any external power supply. We place the permanent magnet on the top of the chip at some distance in such a way that magnetic field is approximately perpendicular to the device surface.

We first obtain the power spectral density of the device when we input a direct current of 100 µA. The external magnetic field is produced by a permanent magnet over the device sited at 23.2 mm. Fig. S1(a) shows the corresponding power spectral density of the device. We observe two different oscillation modes at $f_{a}=1.3$ GHz and at $f_{b}=$ 2.6 GHz.


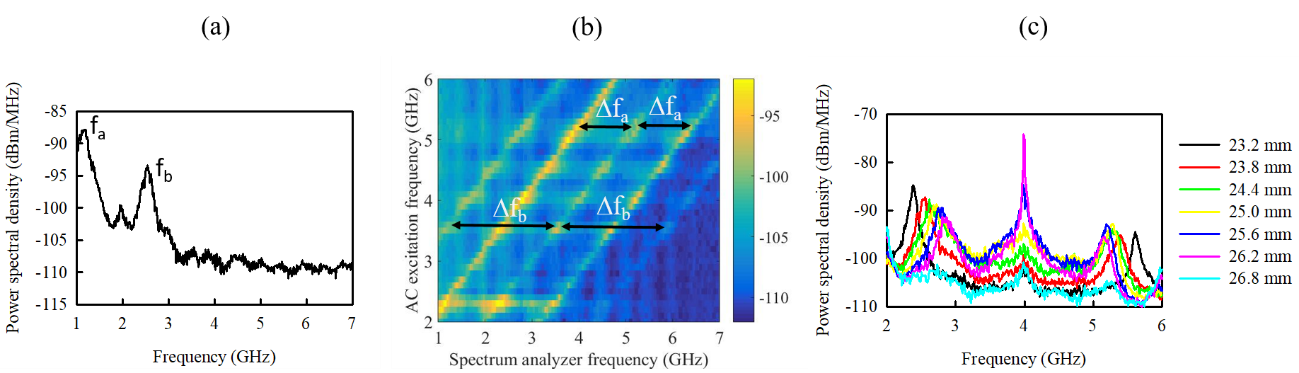


Figure S1. (a) Power spectral density of the device that we employ with permanent magnet when the permanent magnet is over the device at 23.2 mm.

Second, we remove the direct current from the device and we input microwave signal to the solenoid to wirelessly induce microwave current to the device. Fig. S1 (b) shows the power spectral density from the device when we sweep the input signal frequency. Results shows that there are two up-converted and two down-converted branches. The frequency differences $\Delta f_{a}$ and $\Delta f_{b}$ matches to the natural oscillation frequencies $\Delta f_{a}=f_{a}=1.3$ GHz and $\Delta f_{b}=f_{b}=2.6$ GHz. This shows that the two up-converted and the two down-converted branches correspond to mixing between the wirelessly induced microwave signal and the two oscillation modes excited in the magnetic tunnel junction.

Third, we input signal to the solenoid at 4 GHz and moved the permanent magnet to change the external magnetic field. Fig. S1(c) shows the power spectral density when we sweep the distance between the magnet and the device. We observe how the up-converted and the down-converted branches changes depending on the magnetic field position and then, by the magnetic field on the device.
